# Enzymatically synthesized exopolysaccharide of a probiotic strain *Leuconostoc mesenteroides* NTM048 shows adjuvant activity to promote IgA antibody responses

**DOI:** 10.1080/19490976.2021.1949097

**Published:** 2021-07-21

**Authors:** Chiaki Matsuzaki, Yukari Nakashima, Ikuto Endo, Yusuke Tomabechi, Yasuki Higashimura, Saki Itonori, Koji Hosomi, Jun Kunisawa, Kenji Yamamoto, Keiko Hisa

**Affiliations:** a Research Institute for Bioresources and Biotechnology, Ishikawa Prefectural University, Ishikawa Japan; b Laboratory of Vaccine Materials, Center for Vaccine and Adjuvant Research, and Laboratory of Gut Environmental System, National Institutes of Biomedical Innovation, Health and Nutrition, Osaka Japan; c Department of Applied Chemistry, School of Engineering, Tokai University, Kanagawa, Japan; d Department of Food Science, Ishikawa Prefectural University, Ishikawa Japan; e Department of Chemistry, Faculty of Liberal Arts and Education, Shiga University, Shiga Japan; f Center for Innovative and Joint Research, Wakayama University, Wakayama, Japan; g Management Office, Noster Inc, Kyoto, Japan

**Keywords:** *Leuconostoc mesenteroides*, exopolysaccharide, adjuvant, probiotic, glucosyltransferase, IgA

## Abstract

*Leuconostoc mesenteroides* strain NTM048 produces an exopolysaccharide (EPS; glucose polymers 94% and fructose polymers 6%) with adjuvanticity for mucosal vaccination. Strain NTM048 includes three putative EPS-synthesizing genes, *gtf1* and *gtf2* for synthesizing glucose polymers, and *lvnS* for synthesizing fructose polymer. To elucidate the key polymer structure for adjuvanticity, two genes, *gtf1* and *gtf2*, which were annotated as glycoside hydrolase family 70 enzyme genes, were expressed in *Escherichia coli*. Glycosyl-linkage composition analysis and NMR analysis showed that the recombinant enzyme Gtf1 produced a soluble form of α-1,6-glucan, whereas the recombinant enzyme Gtf2 produced glucans with approximately equal percentages of α-1,6- and α-1,3-glucose residues both in the supernatant (S-glucan) and as a precipitate (P-glucan). Comparison of polysaccharides synthesized by Gtf1, Gtf2, and LvnS revealed that Gtf2-S-glucan, which was produced in the supernatant by Gtf2 and formed particles of 7.8 µm, possessed 1.8-fold higher ability to stimulate IgA production from murine Peyer’s patch cells than native NTM048 EPS. Evaluation of adjuvanticity by intranasal administration of mice with an antigen (ovalbumin) and Gtf2-S-glucan or NTM048 EPS showed that Gtf2-S-glucan induced the production of higher antigen-specific antibodies in the airway mucosa and plasma, suggesting a pivotal role of Gtf2-S-glucan in the adjuvanticity of NTM048 EPS.

## Introduction

Exopolysaccharides (EPSs) are polymers synthesized by several bacterial groups during fermentation, which exhibit physiological properties and biological activities. EPS produced by lactic acid bacteria have particularly received intensive interest,^1–3^ mainly because lactic acid bacteria are “generally recognized as safe (GRAS)” for human consumption,^4^ and their EPSs confer health-promoting effects, such as antitumor,^5–7^ antioxidant,^8–10^ cholesterol-lowering,^11–13^ and immunomodulating activities.^14–17^

The functional effects of EPSs are closely related to their chemical structures, but a comprehensive understanding of structure-function relationships between EPS structure and their counterpart properties remains elusive because EPSs vary with the structure of monosaccharide composition, degree of branching from linear molecules, and linkage type between monosaccharides.^3,18^ For effective strategies for the industrial application of EPSs, the chemical structure must be revealed to elucidate their functional properties.

Depending on the composition of their repeating units, EPSs can be classified as homopolysaccharides or heteropolysaccharides. Heteropolysaccharides are synthesized intracellularly via the Wzy-dependent pathway involving several types of glycosyltransferases and have repeating units composed of two or more monosaccharides, commonly glucose, galactose, rhamnose, mannose, and *N*-acetyl-galactosamine. In contrast, homopolysaccharides are synthesized in the extracellular matrix by only one type of extracellular glycosyltransferase and are composed of one monosaccharide: glucose or fructose. Therefore, two types of homopolysaccharides are synthesized: α-glucans by glucosyltransferase belonging to the CAZy^19^ glycoside hydrolase family 70 (GH70) and β-fructans by fructosyltransferase belonging to the CAZy glycoside hydrolase family 68 (GH68).^3,20–22^

We recently demonstrated that the administration of homopolysaccharide-producing *Leuconostoc mesenteroides* strain NTM048 cells or EPS produced by this strain (NTM048 EPS) increased intestinal immunoglobulin A (IgA), which is a crucial mucosal barrier.^15,23^ IgA is secreted onto mucosal surfaces and makes dietary antigens, toxins, and pathogenic microorganisms as targets for attack.^24,25^ *In vitro* treatment of NTM048 EPS to murine Payer’s patch (PP) cells also stimulated IgA production, indicating that IgA-inducing ability of NTM048 EPS is not caused indirectly by prebiotic effects involving intestinal microbiota but rather that it is the result of a direct stimulant against immune cells.^15,26^ Furthermore, nasal administration of NTM048 EPS induced an antigen-specific antibody response, demonstrating the possibility for the use of adjuvants for mucosal vaccination.^16^

Approximately 94% of the NTM048 EPS consists of α-1,6-glucans containing 4% of 1,3-linked α-glucose branches and the remaining consists of β-fructans.^16^ Draft genome sequencing of strain NTM048 revealed that it contained two putative GH70 genes and a single putative GH68 gene (*lvnS*).^27^ We already synthesized β-fructans using the recombinant LvnS expressed by *Escherichia coli*. However, the IgA-inducing activity of the β-fructans was not higher than that of NTM048 EPS,^27^ suggesting that α-glucans, which were synthesized by GH70 enzymes, contribute greatly to the induction of IgA. In general, GH70 enzymes catalyze the polymerization of α-glucans, usually using sucrose as the glucose donor and transferring this glucose residue to the non-reducing end of the growing glucan.^28,29^ Moreover, GH70 enzymes can synthesize α-glucans, which vary in terms of size, glycosidic linkages, and the degree and arrangement of branching.^30^ In this study, to ascertain the functional chemical structure of NTM048 EPS to stimulate IgA production, we cloned GH70 enzymes from strain NTM048 and synthesized α-glucans, which are a major component of NTM048 EPS. We also cloned GH70 enzymes from *L. mesenteroides* strain JCM6124, which is the type strain of *L. mesenteroides* harboring lower-IgA inducing activity than strain NTM048.^31^ Moreover, we evaluated the IgA-inducing ability of these synthesized glucans *in vitro* and investigated the adjuvant activity to promote antigen-specific IgA responses *in vivo*. Through these studies, we attempted to clarify the structure–function relationship of NTM048 EPS.

## Results

### Gene analyses of GH70 enzymes of strain NTM048

**Figure 1.**
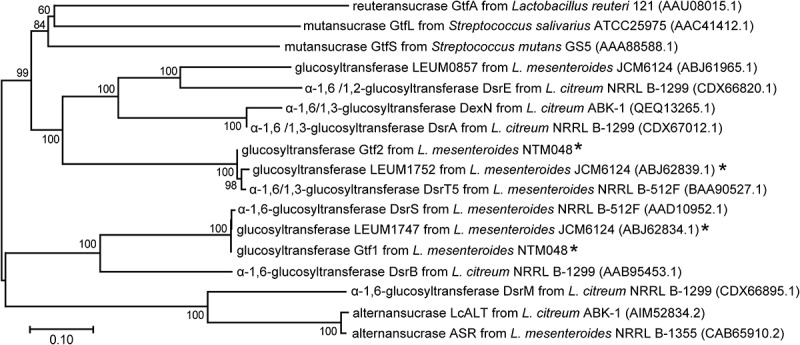
Phylogenetic analysis of glucosyltransferases derived from strains NTM048 and JCM1624 and typical glucosyltransferases for which the structural features of synthesized α-glucans are available. Each protein is labeled with its GenBank accession number. The dendrogram was generated using the neighbor-joining method using MEGA X software. Bootstrap values (expressed as percentages of 1,000 replications) are given at branching points. Bar 10% sequence divergence. * Enzymes used for this study

**Figure 2.**
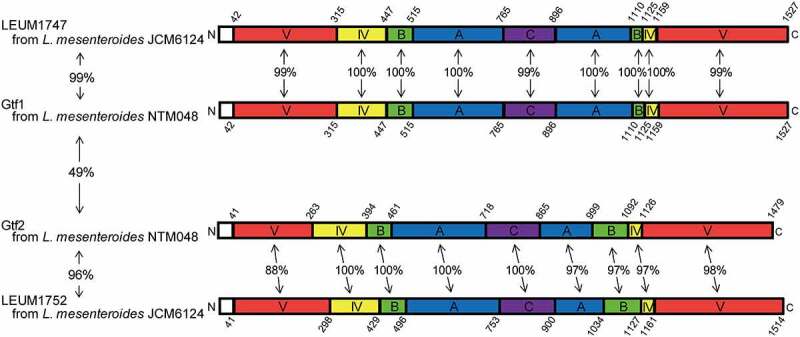
Schematic representation of the domain organization of Gtf1, LEUM1747, Gtf2, and LEUM1752. Numbering after N-terminal signal peptide (white) corresponds to residues delimiting the different domains (red, domain V; yellow, domain IV; green, domain B; blue, domain A; purple, domain C) predicted by alignment with DSR-M.^34^ The percentage represents the similarity of amino acid sequences between domains or enzymes

The draft genome sequence of strain NTM048 included two open reading frames annotated to glucosyltransferase genes belonging to the GH 70 enzyme, which catalyzes the synthesis of α-glucans. The phylogenetic analysis of the glucosyltransferase genes is shown in Figure 1. One gene, designated as *gtf1*, is 4,584 bp in length and encodes a protein of 1,527 amino acid residues. The amino acid sequence of the *gtf1* protein, designated as Gtf1, had 99% identity with LEUM1747 (GenBank ABJ62834.1) derived from the type strain *L. mesenteroides* JCM6124 and 99% identity with DsrS derived from *L. mesenteroides* NRRL B512-F, which synthesizes α-1,6-glucan.^32^ The other gene, designated as *gtf2*, is 4,440 bp in length and encodes a protein of 1,479 amino acid residues. The amino acid sequence of the *gtf2* protein, designated as Gtf2, had 96% identity with LEUM1752 (GenBank ABJ62839.1) derived from the type strain JCM6124. Moreover, Gtf2 shared 95% identity with DsrT5 derived from *L. mesenteroides* NRRL B-512 F, which synthesizes insoluble α-glucans with 1,6- and 1,3-linkages.^33^ The known enzyme structure of GH70 enzymes adopts a “U-shaped” folding and is organized into five domains: domains A, B, C, IV, and V.^29,34^ To gain additional insight into the structural differences, we predicted the domain structures of Gtf1, LEUM1747, Gtf2, and LEUM1752 by alignment amino acid sequences with DSR-M,^34^ of which the most complete domain structure was solved (Figure 2). Comparing amino acid sequences between Gtf2 and LEUM1752, the most different region was domain V, which was located at the N-terminal and C-terminal extremity and which participates in the lengthening of the product by holding growing α-glucan from the catalytic domain (domain A).^34^ In contrast, almost no difference between Gtf1 and LEUM1747 was found though the sequences of Gtf1 and Gtf2 were 49% identical. Consistent with the extracellular location of GH70 enzymes, the protein sequences of Gtf1, Gtf2, LEUM1747, and LEUM1752 contained a typical gram-positive signal peptide, which was predicted using the SignalP server (http://www.cbs.dtu.dk/services/SignalP-4.1/).

**Figure 3.**
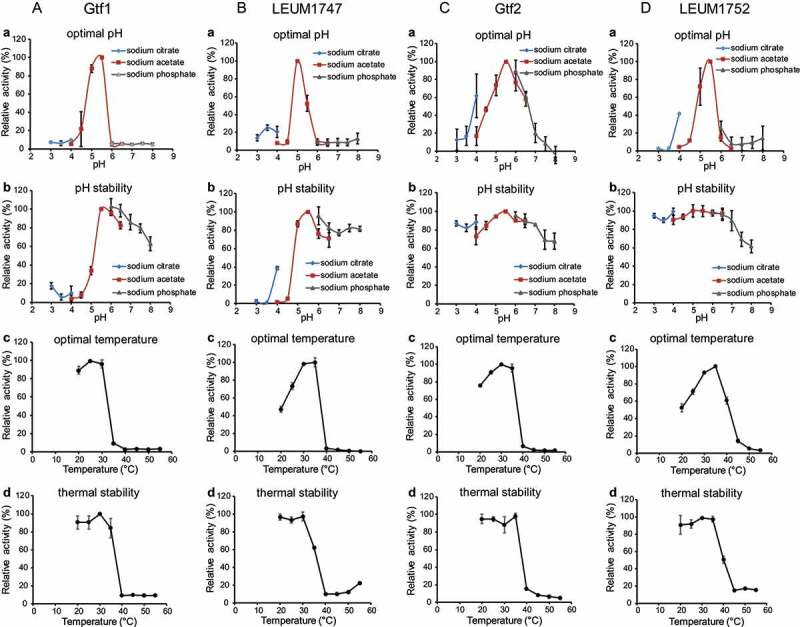
Enzymatic characterization of Gtf1 (A), LEUM1747 (B), Gtf2 (C), and LEUM1752 (D). Optimal pH (a) and pH stability (b) of these enzymes were evaluated in 20 mM sodium citrate (pH 3.0–4.0), 20 mM sodium acetate (pH 4.0–6.5), and 20 mM sodium phosphate (pH 6.0–8.0) at 28°C. Optimal temperature (c) and thermal stability (d) of these enzymes were assayed at 20–55°C in 20 mM sodium citrate buffer (pH 5.5)

Based on the nucleotide sequence information, genes encoding glucosyltransferases, Gtf1, Gtf2, LEUM1747, and LEUM1752 were cloned and expressed in *E. coli*. Enzymes were purified using affinity chromatography and anion-exchange chromatography (Figure S1), and their activities were evaluated (Table 1). The specific activities of purified Gtf1, LEUM1747, Gtf2, and LEUM1752 were 16.9, 13.3, 24.1, and 9.3 units/mg, respectively (Table 1), showing similar activity reported for other GH70 enzymes (13.9 units/mg^35^ or 29.3 units/mg^36^). As shown in Figure 3, glucosyltransferases (A, Gtf1; B, LEUM1747; C, Gtf2; and D, LEUM1752) had similar enzymatic properties, showing an optimal pH from 5.0 to 5.5 (Figure 3a) and an optimal temperature of around 30°C (Figure 3c). A similar observation was also reported for GH70 enzyme: DSRBCB4 from *L. mesenteroides* B1299CB4 at pH 5.2 and 30°C^35^ and dextransucrase from *L. mesenteroides* B-512 FM at pH 5.2 and 28°C.^37^ Furthermore, thermal stability was achieved up to 35°C, exhibiting the same profile (Figure 3d). However, although the activity of Gtf1 and LEUM1747 decreased under pH 5.0 (Figure 3Ab, Bb), Gtf2 and LEUM1752 were stable over a wide range of pH values (Figure 3Cb, Db).

**Table 1.** Kinetic parameters and specific activities of recombinant glucosyltransferases Gtf1, Gtf2, LEUM1747, and LEUM1752

	*K*_m_ (mM)	*k*_cat_/*K*_m_ (M^−1^・sec^−^^1^)	specific activity (units・mg^−^^1^)
Gtf1	2.5 × 10^1^ ± 0.2	1.8 × 10^3^ ± 0.2	1.7 × 10^1^ ± 0.2
LEUM1747	2.5 × 10^1^ ± 0.2	1.4 × 10^3^ ± 0.1	1.3 × 10^1^ ± 0.0
Gtf2	2.3 × 10^1^ ± 0.3	2.8 × 10^3^ ± 0.3	2.4 × 10^1^ ± 0.1
LEUM1752	1.9 × 10^1^ ± 0.1	1.3 × 10^3^ ± 0.0	9.3 ± 0.2

### Production and characterization of glucans

**Figure 4.**
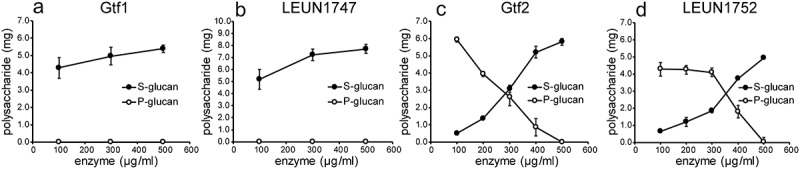
Glucan production using Gtf1 (a), LEM1747 (b), Gtf2 (c), and LEUM1752 (d) in 50 mM sucrose as substrate. After incubation for 24 h at 28°C, reaction solutions were centrifuged, and glucans in the supernatant (S-glucan) and precipitated glucans (P-glucan) were measured

Glucans were synthesized in 50 mM sucrose using various concentrations of purified glucosyltransferases. After centrifugation (8,000 × *g*, 10 min), glucans in the supernatant (S-glucan) and precipitated glucans (P-glucan) were examined. When glucans were produced by Gtf1 or LEUM1747 at concentrations of 100, 300, and 500 µg/ml, no precipitate was observed, and glucan was detected in the supernatant (4.3, 5.0, and 5.4 mg/ml, respectively, for Gtf1 or 5.2, 7.2, and 7.7 mg/ml, respectively, for LEUM1747) (Figure 4a,b). The molecular masses were 3.1 × 10^6^ for Gtf1 and 4.5 × 10^6^ for LEUM1747 (Table 2). When glucan production was performed by Gtf2 or LEUM1752, the solubility of the produced glucans depended on the enzyme concentration (Figure 4c,d). When 100 µg/ml of Gtf2 was used, synthesized glucans were precipitated (P-glucan) by centrifugation, and the concentration of glucan in the supernatant (S-glucan) was less than 0.3 mg/ml. However, 400 µg/ml of Gtf2 synthesized both S-glucan and P-glucan (5.2 and 0.87 mg/ml, respectively), and 500 µg/ml of Gtf2 synthesized only S-glucan (5.8 mg/ml), with a molecular mass of 14.4 × 10^6^ (Table 2). In addition, LEUM1752 (500 µg/ml) synthesized only S-glucans (Figure 4d), but the molecular mass was 25.7 × 10^6^, which was higher than that of Gtf2 (Table 2).

**Table 2.** Molecular mass, particle size, and linkage analyses of various glucans produced from sucrose by recombinant glucosyltransferases Gtf1, Gtf2, LEUM1747, and LEUM1752

Glucan	Molecular mass(×10^6^)	Particle size(µm)	Glucopyranose methylation (%)			
			T-Glc*p*-(1→	→3-Glc*p*-(1→	→6-Glc*p*-(1→	→3,6-Glc*p*-(1→
Gtf1 (S-glucan)*	3.1	0.035 ± 0.007, 0.294 ± 0.028	9	N/A	81	10
LEUM1747 (S-glucan)*	4.5	0.070 ± 0.027, 0.386 ± 0.065	8	N/A	77	12
Gtf2 (S-glucan)**	14.4	7.8 ± 0.4	8	43	45	4
Gtf2 (*P*-glucan)*	-	-	6	44	39	11
LEUM1752 (S-glucan)**	25.7	12.3 ± 1.5	13	32	49	6
LEUM1752 (*P*-glucan)*	-	-	8	51	36	5

*Glucan produced using 100 µg/ml enzyme, **Glucan produced using 500 µg/ml enzyme.

**Figure 5.**
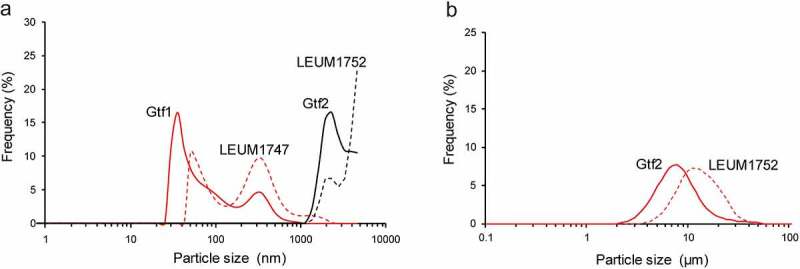
Particle size distributions of S-glucans. (a) S-glucans from Gtf1, Gtf2, LEUM 1747, and LEUM 1752 were measured using NanotracWaveII. (b) S-glucans from Gtf2 and LEUM 1752 were measured using MT3300EX II

These purified glucans showed different physicochemical properties. S-glucans synthesized by Gtf1 or LEUM1747 (100 µg/ml enzymes were used) dissolved freely in the water. S-glucans synthesized by 500 µg/ml Gtf2 or LEUM1752 formed small particles and dispersed in water, whereas P-glucans synthesized by 100 µg/ml of Gtf2 or LEUM1752 were deposited at the bottom. When the particle size distributions were analyzed using NanotracWaveII, two peaks were found in the S-glucans of Gtf1 and LEUM1747: 35 and 294 nm for Gtf1 and 70 and 386 nm for LEUM1747 (Figure 5a, Table 2). In contrast, the particle sizes of S-glucans from Gtf2 and LEUM1752 were analyzed using MT3300EX II because the particles of S-glucans of Gtf2 and LEUM1752 were larger than 1.0 µm (Figure 5a). The peak of each glucan was found at 7.8 µm for Gtf2 and 12.3 µm for LEUM1752 (Figure 5b, Table 2).

To elucidate the linkage pattern of glucan samples, we prepared partially methylated alditol acetate derivatives and analyzed them using GC-MS (Table 2). S-glucans from Gtf1 and LEUM1747 harbored 6-linked glucose units in a polymer backbone and exhibited 10–12% branching with 3,6-linked residues. On the other hand, 3-linked and 6-linked glucose with 3,6-linked branching residues were detected in both S- and P-glucans from Gtf2 and LEUM1752, but the 3-linkage/6-linkage ratio was different. For S-glucan, the ratio of 3-linkage was lower than that of 6-linkage, whereas the former was higher than the latter in P-glucan. Glucans mainly harboring α-1,3-linked glucose are generally water-insoluble, whereas glucans consisting of a high level of α-1,6-linked glucose are soluble in water.^36,38,39^ A high ratio of 1,3-linked glucose might cause precipitation of P-glucans.

**Figure 6.**
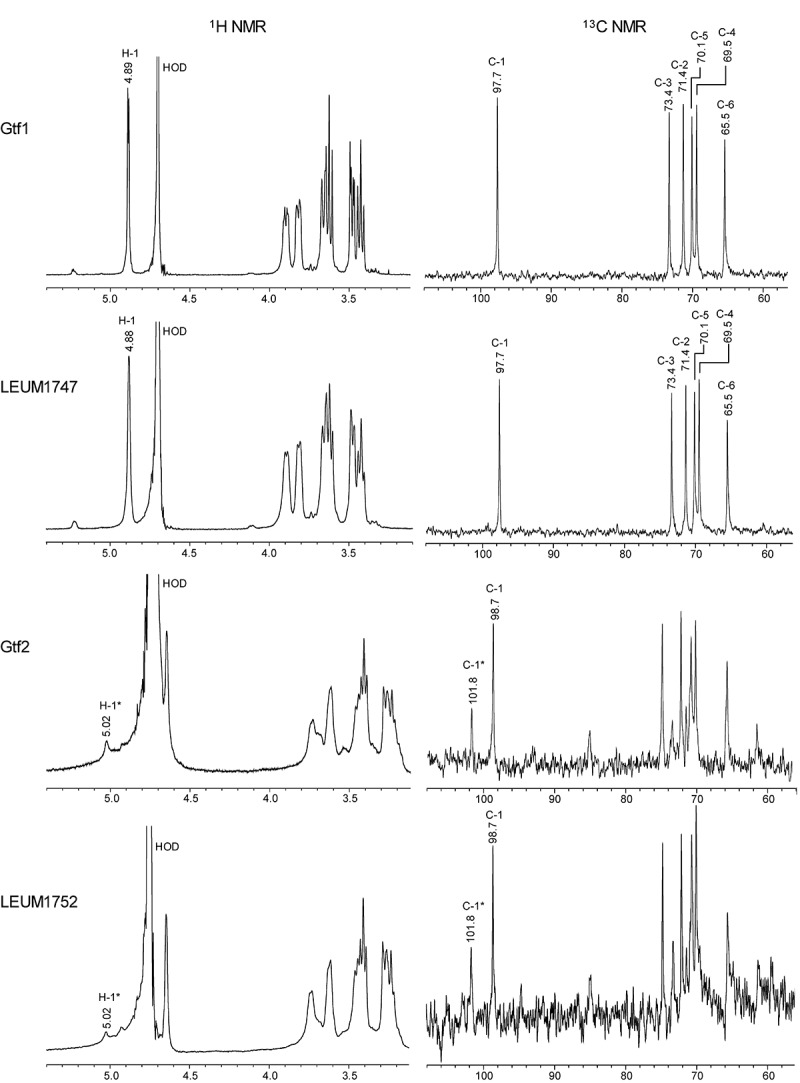
The^1^H NMR and^13^C NMR spectra of glucans. S-glucans from Gtf1 and LEUM1747 were analyzed in D_2_O and S-glucans from Gtf2 and LEUM1752 were analyzed in NaOD-D_2_O. Signals corresponding to α-1,3-linked residues are labeled with *

The^1^H NMR and^13^C NMR spectra of S-glucans of Gtf1 and LEUM1747 showed a single set of signals (Figure 6). These spectra showed good agreement with literature data for long α-1,6-glucans.^32,36^ The respective positions of anomeric signals at 4.89 and 97.7 ppm for S-glucan of Gtf1 or 4.88 and 97.7 ppm for S-glucan of LEUM1747 in the^1^H and^13^C NMR spectra, respectively, are characteristic of α-1,6-glucosidic linkages. The^1^H NMR and^13^C NMR spectra of S-glucans of Gtf2 and LEUM1752 indicated the presence of both α-1,6- and α-1,3-glucosidic chains. In the^1^H NMR spectra of S-glucans of Gtf2 and LEUM1752, although the anomeric proton signal of the α-1,6-glucosyl unit was hidden under the HOD signal, the chemical shift of 5.02 ppm can be assigned to anomeric protons of α-1,3-glucosyl unit.^36^ In the^13^C NMR spectra of S-glucans of Gtf2 and LEUM1752, the positions of anomeric signals at 101.8 and 98.7 ppm are characteristic of α-1,3- and α-1,6-glucosidic linkages, respectively.^36^

### In vitro analysis of IgA-inducing activity of glucan samples

**Figure 7.**
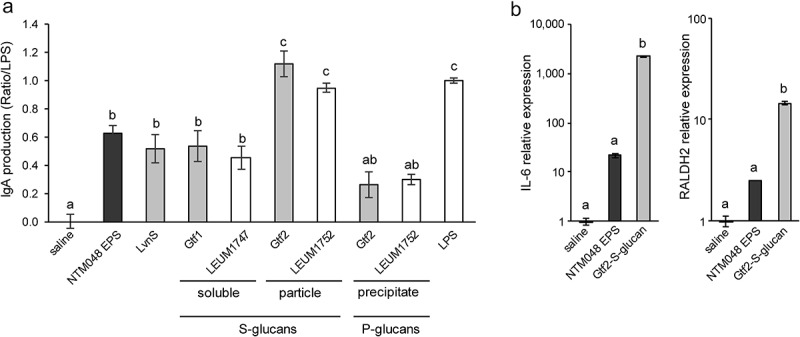
Evaluation of IgA-inducing activity *in vitro*. (a) Comparison of IgA-inducing activities of glucans, fructan, and NTM048 exopolysaccharide (EPS). Black bar: NTM048 EPS. Gray bar: polysaccharides synthesized by strain NTM048 enzymes (fructan synthesized by LvnS and glucans synthesized by Gtf1 and Gtf2). White bar: polysaccharides synthesized by strain JCM6124 enzymes (glucans synthesized by LEUM1747 and LEUM1752); lipopolysaccharide (LPS) was used as a positive control. Each value is presented as means ± SEM (*n* = 6). (b) Gene expression analysis of bone marrow-derived DCs (BMDCs) stimulated with NTM048 EPS or S-glucan from Gtf2 (Gtf2-S-glucan). The values represent the difference between the stimulated cells and the non-stimulated control cells. Each value is presented as means ± SEM (*n* = 3–4). Different letters (a-c) show significant differences (*P* < .05)

We previously reported that NTM048 EPS could stimulate IgA production from PP cells of mice.^15,23^ To reveal the component of NTM048 EPS to contribute IgA production, we evaluated the IgA-inducing activity of glucans synthesized by glucosyltransferases and compared them to those of native NTM048 EPS and fructan synthesized by LvnS derived from strain NTM048.^27^ Before testing the treatments on PP cells with polysaccharides, we investigated any cytotoxic effect on the cells by examination of the number of viable PP cells. After incubation with polysaccharide samples (250 µg/ml) for 5 days, the number of viable PP cells was 95–104% of that after incubation with saline control, whereas the number of viable PP cells after incubation with lipopolysaccharide (LPS; 5 µg/ml) was 92% of that after incubation with saline control. These results suggested that the polysaccharide samples almost not exhibit cytotoxicity. As shown in Figure 7a, all polysaccharide samples possessed IgA-inducing activity, but their levels of activity were different. The IgA-inducing activities of fructan and S-glucan synthesized by Gtf1 were slightly lower than that of NTM048 EPS, but the differences were not significant. The IgA-inducing activity of S-glucan synthesized by Gtf2 was 1.8-fold higher than that of NTM048 EPS. The activity of P-glucans synthesized by Gtf2 was one-third that of NTM048 EPS. Among polysaccharides synthesized by enzymes that catalyze NTM048 EPS synthesis, S-glucan synthesized by Gtf2 might contribute greatly to the IgA-inducing activity of NTM048 EPS. The IgA-inducing activity of glucan samples might depend on the glucan type and not on the source strains of the organisms that synthesize glucans. S-glucan from Gtf2 showed higher activity (1.2-fold) than that of S-glucan from LEUM1752, but not significantly higher. The differences in the molecular size, the ratio of 1,3-linkage/1,6-linkage of glucose residues or the particle size (Table 2) of glucans might affect the IgA-inducing activity (see discussion).

In the previous study, we demonstrated that NTM048 EPS stimulated dendritic cells to express genes of IL-6 and retinal dehydrogenase 2 (RALDH2) that promote mucosal IgA production (see discussion).^26^ To evaluate the effect on dendritic cells, murine bone marrow-derived dendritic cells (BMDCs) were cultured and stimulated with NTM048 EPS or S-glucan from Gtf2 (Gtf2-S-glucan); changes in the expression of IL-6 and RALDH2 genes as the mediators of mucosal IgA expression were analyzed (Figure 7b). Gtf2-S-glucan stimulation induced significantly higher gene expressions of IL-6 (99-fold) and RALDH2 (5.7-fold) than those by NTM 048 EPS, suggesting that stronger stimulation of dendritic cells by Gtf2-S-glucan resulted in a higher IgA production from PP cells.

### In vivo adjuvant activity of the glucan samples

**Figure 8.**
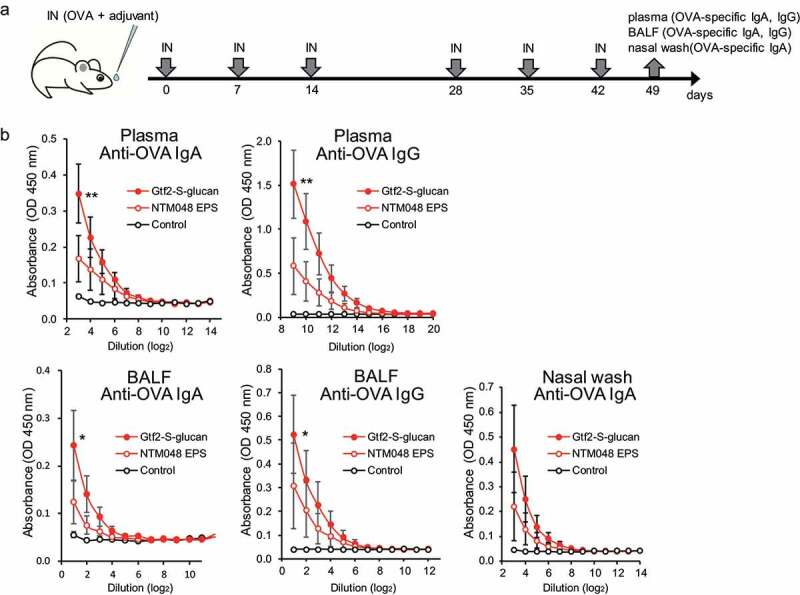
Induction of ovalbumin (OVA)-specific systemic and respiratory antibody responses by intranasal immunization with OVA + Gtf2-S-glucan. (a) Time schedules for intranasal vaccination (IN) and the sampling of blood, BALF, and nasal washes. Each dose contained 5 µg of OVA alone (control) or 5 µg of OVA with 100 µg of glucan (NTM048 EPS or Gtf2-S-glucan). (b) On day 49, the OVA-specific antibodies in plasma, BALF, and nasal washes were measured. Data are from individual mice and represent the mean ± SEM (*n* = 7–8). Tukey–Kramer multiple comparison test. *P < .05, **P < .01 vs. control

Next, we investigated whether Gtf2-S-glucan is more effective than NTM048 EPS in inducing ovalbumin (OVA)-specific antibody responses. All animals used in the experiment were in good health throughout the experimental period. No significant differences were found in body weight gain among the groups. One week after the last immunization, plasma, bronchoalveolar lavage fluid (BALF), and nasal wash were collected for enzyme-linked immunosorbent assay (ELISA) to measure the production of OVA-specific antibodies (Figure 8a). Figure 8b shows that mice nasally immunized with Gtf2-S-glucan induced significantly higher OVA-specific plasma IgA and IgG than the control mice (*P* < .01). In addition to the systemic immune compartment, mice nasally immunized with Gtf2-S-glucan showed higher levels of OVA-specific IgA and IgG in the BALF (*P* < .05) and IgA in the nasal washes (*P* = .08). These findings indicate that OVA-specific antibody responses were induced in the respiratory tract and systemic immune compartments. Although no statistically significant difference was found between Gtf2-S-glucan and NTM048 EPS, the levels of OVA-specific IgA and IgG in plasma and BALF and OVA-specific IgA in the nasal wash, which were induced by Gtf2-S-glucan, were higher than those induced by NTM048 EPS. Gtf2-S-glucan is expected to be more effective than NTM048 EPS in enhancing the mucosal immune response to antigens.

## Discussion

GH70 enzymes produce α-glucans, which have different linkage compositions, branching degrees, and size distributions. In this study, the amino acid sequences of Gtf1 and LEUM1747 showed 99% identity with DsrS derived from *L. mesenteroides* NRRL B512-F (Figure 1), which synthesizes soluble α-1,6-linked glucans.^32^ Gtf1 and LEUM1747 were found to synthesize soluble α-1,6-linked glucans, agreeing with the high similarity of amino acid sequence with DsrS. On the other hand, Gtf2 and LEUM1752 showed 96% and 98% identity, respectively, with DsrT5 derived from *L. mesenteroides* NRRL B-512 F, which synthesizes insoluble α-glucan with 1,6- and 1,3-linkage.^33,40^ Both Gtf2 and LEUM1752 were found to synthesize glucans composed of α-1,6- and α-1,3-linked glucose, but their molecular sizes were not identical. Glucan produced by LEUM1752 was about twice as larger in terms of molecular size than that produced by Gtf2. Among the five domains (domains A, B, C, IV, and V) forming the GH70 enzyme, domain V is called the glucan-binding domain, which contributes to the polymerization of glucan.^34^ The amino acid sequences of domain V were the most different in the full amino acid sequences of Gtf2 and LEUM1752 (Figure 2). The difference in the molecular size of glucans produced by both enzymes may be attributed to the difference in domain V.

Although EPSs have been reported to modulate the immune system, the results of many studies have suggested that the EPS structure might affect the immune response. To date, in attempts to correlate some physicochemical traits of EPS with their immune-modulating capacity, two traits have been proposed.^2,41^ First, acidic EPSs, which are characterized by the presence of phosphate, are good inducers of immune responses. For example, *Lactobacillus delbrueckii* subsp. *bulgalicus* OLL-1073-R1 synthesized EPS composed of two fractions: acidic and neutral EPSs. Both EPSs contained glucose and galactose residues, but only acidic EPS had 0.1% PO_4_^−^ and stimulated the immune system.^42^ Although the role of PO_4_^−^ in immune stimulation was also proven by the fact that EPS synthesized by *L. mesenteroides* was chemically phosphorylated and the resultant EPS augmented cytokine expressions,^43^ natural homopolysaccharide EPSs synthesized by *L. mesenteroides* were not modified with phosphate.^44^ In this study, phosphate was not detected in the enzyme-synthesized glucans (data not shown). The other trait associated with the immune properties of EPS was the polymer size.^45^ EPSs with high molecular weight are proposed to reduce the production of inflammatory cytokines and induce immune homeostasis.^41,45^ Bleau et al.^46^ demonstrated that high-molecular-weight EPS (5.3 × 10^5^ Da) obtained from *Lacticaseibacillus rhamnosus* RW-9595 M could suppress inflammatory cytokine production from macrophages by the induction of anti-inflammatory IL-10. Furthermore, among EPSs purified from three closely related *Bifidobacterium animalis* subsp. *lactis* strains, only the high-molecular-weight EPS (approx. 10^6^ Da) suppressed immune response from peripheral blood mononuclear cells.^47^ In our study, glucans with high molecular weight and large particle size from Gtf2 and LEUM1752 (15–25 × 10^6^ Da, 7.8–12.3 µm) elicited stronger IgA induction than glucans with low molecular weight and small particle size from Gtf1 and LEUN1747 (approximately 5.0 × 10^6^ Da and 0.5 µm, respectively). This result demonstrated the association of molecular weight and particle size of glucan with its expression of immune properties. Some approaches must be attempted to elucidate the most appropriate molecular weight and particle size of glucans.

**Figure 9.**
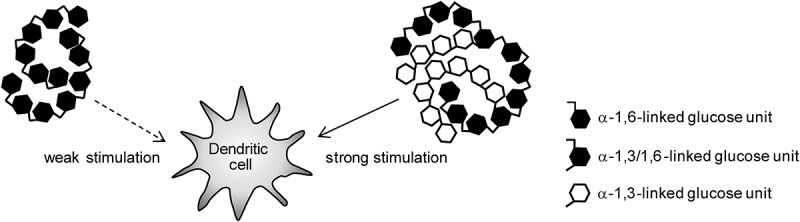
Schematic representation of possible structures to interact with dendritic cells

Phagocytosis is a principal component of the body’s innate immune response in which antigen-presenting cells internalize large (>0.5 µm) particulate targets.^48^ The cellular internalization of particles via phagocytosis involves attractive forces, such as van der Waals, electrostatic, ionic, hydrophilic, and hydrophobic interactions, between the cells and particles.^49^ α-1,3-glucan is known as a bacterial cell wall component that contribute to cell adhesion by hydrophobic interaction.^50,51^ The α-1,3-linked glucose component of Gtf2-S-glucan might play an important role in adhering to antigen-presenting cells. Using murine bone marrow-derived dendritic cells, we previously reported that NTM048 EPS increased the gene expression levels of IL-6, which plays a crucial role in the differentiation of IgA B cells,^24–26^ and RALDH2, which catalyzes retinal to form retinoic acid and stimulates antigen-specific antibody production through thymic stromal lymphopoietin.^26,52^ Furthermore, in this study, we could obtain a suggestion that the enhanced interaction by α-1,3-glucan between Gtf2-S-glucan and dendritic cells might induce stronger adjuvanticity than NTM048 EPS (Figure 9).

In summary, we enzymatically synthesized polysaccharide with adjuvanticity using EPS-synthesizing enzyme derived from the probiotic strain NTM048. The synthesized polysaccharide, Gtf2-S-glucan, is a high molecular weight particle forming α-1,3/1,6-linked glucan. The synthesized polysaccharide possessed stronger adjuvanticity than NTM048 EPS. The bioactivity of EPS is generally strain-dependent, and the obscure activity of EPS is one of the bottlenecks for its application. In this study, we showed a strong association between the bioactivity of EPS and EPS-synthesizing enzymes. Our enzymatic approach to elucidate the structure–function relationships between EPS structure and its biological function is expected to promote the industrial application of EPS as a novel ingredient in functional foods.

## Materials and methods

### Bacterial strains and culture conditions

The EPS producer, *L. mesenteroides* subsp. *mesenteroides* strain NTM048 isolated from fresh green pea and stored in our laboratory, was used for this study.^23^ *L. mesenteroides* subsp. *mesenteroides* JCM6124 was obtained from the Japan Collection of Microorganisms (Saitama, Japan). *Leuconostoc* strains were grown in MRS broth (Becton, Dickinson and Co., Sparks, MD, USA) at 30°C in a stationary culture. *E. coli* strain BL21 (DE3) was cultivated in lysogeny broth (LB; Difco Laboratories, Detroit, MI, USA).

### DNA preparation

Strain NTM048 cells were harvested from the overnight MRS broth by centrifugation (2 min at 4°C at 17,000 × *g*) and washed with a sucrose-ethylenediamine-tetraacetic acid (EDTA) buffer (450 mM sucrose, 1 mM EDTA in 5 mM Tris-HCl [pH 8.0]). The washed cells were resuspended in the same buffer containing 5 µg/ml lysozyme (Sigma Aldrich Corp., St. Louis, MO, USA) and 40 U/ml mutanolysin (Sigma Aldrich Corp.). After incubation for 1 h at 37°C, total DNA was extracted using the Wizard Genomic DNA Purification Kit (Promega, Charbonnières, France).

### Phylogenetic analysis

The draft genome sequence of the *L. mesenteroides* subsp. *mesenteroides* strain NTM048 (GenBank Accession No. BOPR01000001) was determined using the Miseq system (Illumina Inc., San Diego, CA, USA) under the auspices of the Dragon Genomics Center (Takara Bio Inc., Otsu, Japan). The genome sequence was analyzed using *in silico* Molecular Cloning (*In Silico* Biology, Inc., Kanagawa, Japan). The genome included two open reading frames (Leu048_16720 and Leu048_16680) annotated to the enzyme genes belonging to GH70. These GH70 genes were designated as *gtf1* for Leu048_16720 and *gtf2* for Leu048_16680. The amino acid sequences of GH70 enzymes of strain NTM048 were aligned with those of various GH70 proteins using ClustalW^53^ and were employed to build a phylogenetic tree. Evolutionary analyses were conducted in MEGA X.^54^ The evolutionary history was inferred using the neighbor-joining method.^55^ Bootstrap analysis^56^ based on 1,000 replications was applied for evaluating the tree topologies.

### Gene cloning and enzyme expression of glucosyltransferase genes

Gene fragments of *gtf1* and *gtf2* from strain NTM048, and LEUM1747 (GenBank ABJ62834.1) and LEUM1752 (GenBank ABJ62839.1) genes from strain JCM6124 were amplified from genomic DNA by high-fidelity PCR using PrimeSTAR Max (Takara Bio Inc.), following genetic removal of the signal peptide. The primer pairs used for PCR are listed in Table S1. The purified PCR products were inserted into NdeI-Not1-digested pET23b (Novagen Inc., Madison, WI, USA) using the In-Fusion HD PCR Cloning Kit (Clontech Laboratories, Inc., Palo Alto, CA, USA) to produce C-terminal His6-tagged proteins. Protein-expressing plasmids were transformed into *E. coli* BL21 (DE3) cells. The transformants were cultured in LB medium supplemented with 100 μg/ml ampicillin. After induction with 0.1 mM isopropyl-β-D-thiogalactopyranoside (IPTG), the transformants were cultured at 20°C for 20 h and were harvested by centrifugation (30 min at 4°C at 8,000 × *g*). After the pellet was washed with 150 mM NaCl in 20 mM phosphate buffer (pH 7.5), the suspension was centrifuged again (30 min at 4°C at 8,000 × *g*). The pelleted cells were resuspended in 150 mM NaCl in 20 mM phosphate buffer (pH 7.5) and lysed by sonication (Qsonica LLC, Newton, CT, USA), followed by centrifugation (15 min at 4°C at 17,000 × *g*). The clear cell lysate of the supernatant was loaded onto a Ni-NTA column (HisTrap HP; GE Healthcare, Uppsala, Sweden). Binding was achieved using 20 mM sodium phosphate buffer (pH 7.5) containing 0.5 M NaCl and 20 mM imidazole, followed by washing with the same buffer. The His-tagged proteins were eluted using 20 mM sodium phosphate buffer (pH 7.5) containing 0.5 M NaCl and 500 mM imidazole. The eluted fraction was dialyzed against 20 mM Tris-HCl buffer (pH 8.0) and purified further by Hi-Trap Q HP (GE Healthcare) column chromatography (0–1.0 M NaCl in 20 mM Tris-HCl buffer [pH 8.0]). The protein concentration was determined using a BCA Protein Assay Kit (Thermo Scientific, Rockford, IL, USA) with bovine serum albumin as a standard.

### Kinetic analysis and characterization of recombinant enzymes

Kinetic analysis of the enzymes was conducted in 20 mM sodium acetate buffer (pH 5.5) containing 5–200 mM sucrose and 1 mM CaCl_2_ at 28°C. The enzymatic activity was measured by determining the fructose released from 50 mM sucrose in 20 mM sodium acetate buffer (pH 5.5) containing 1 mM CaCl_2_ at 28°C using the 3,5-dinitrosalicylic acid (DNS) method.^35,57^ One glucosyltransferase unit was defined as the amount of enzyme that produces 1 µmole of fructose from the substrate sucrose per minute. The reaction was terminated by adding 50 mM NaOH.

The effect of pH on the enzyme activity was determined by varying the pH between 3.0 and 8.0. The buffers used were sodium citrate (pH 3.0–4.0), sodium acetate (pH 4.0–6.5), and sodium phosphate (pH 6.0–8.0). To determine the optimum pH, the enzymes (10–50 µg/ml) were incubated with 50 mM sucrose and 1 mM CaCl_2_ in 20 mM buffer at various pH values. The optimal temperature was determined by incubating the reaction mixture containing 50 mM sucrose and 1 mM CaCl_2_ in 20 mM sodium acetate buffer (pH 5.5) at various temperatures for a short period of time (<1 min). The temperature and pH stabilities were examined, respectively, by measuring the residual activity following enzyme incubation (10–50 µg/ml) with 50 mM sucrose in 20 mM sodium citrate buffer (pH 5.5) for 30 min at various temperatures and overnight incubation of enzymes (10–50 µg/ml) in various buffers (20 mM) at 4°C.

### Production of glucans by recombinant enzymes and molecular weight measurement

Glucans were synthesized using GH70 recombinant enzymes in 50 mM sucrose, 1 mM CaCl_2_, and 100 mM sodium acetate (pH 5.5) at 28°C for 24 h. After confirming the depletion of sucrose using thin-layer chromatography (Silica gel 60; Merck, Tokyo, Japan) with chloroform-methanol-water (3:3:1 v/v/v), the reaction was terminated by incubation at 100°C for 20 min. After centrifugation (10 min at 8,000 × *g*), the molecular weight distributions of glucans in the supernatant were determined by high-performance size-exclusion chromatography with Shodex OHpak SB-807 G (Guard), SB-807 HQ, and SB-806 M HQ (8.0 mm ID × 300 mm length; Showa Denko KK, Tokyo, Japan) at 40°C and estimated using dextran standards (150, 270, and 670 kDa from Sigma Aldrich Corp., 3,755 kDa from American Polymer Standards Corp., Mentor, OH, USA). The amounts of synthesized glucans were also calculated using dextran 150 kDa as the standard (Sigma–Aldrich Corp.). Samples (injected volume: 20 µl) were eluted using 0.3 M NaNO_3_ at a flow rate of 1 ml/min and were detected using a refractive index (RI) detector RID10 (Shimadzu Corp., Kyoto, Japan). Glucans in the supernatant were purified by size-exclusion chromatography using Sepharose CL6B (Sigma–Aldrich Corp.) and were freeze-dried. Precipitated glucans were purified by washing with MilliQ water twice and were dried in vacuum. The absence of protein in the glucan sample was confirmed by measuring the absorbance at 280 nm.

### Particle size distribution

Particle sizes were analyzed using laser scattering particle size distribution analyzers of Nanotrac Wave II (when smaller than 1.0 µm, MicrotracBEL Corp., Osaka, Japan) and Microtrac MT3300EXII (when larger than 1.0 µm, MicrotracBEL Corp.). The distribution was measured based on length distributions using 1.60 as a relative refractive index. The mode size, which is the most common size of the particles in the population, was determined for each formulation. The data are the averages of three measurements for each sample.

### NMR analysis

The NMR spectra (500 MHz, AVANCE III HD; Bruker Analytik GmbH, Rheinstetten, Germany) were recorded using approximately 5 mg of polysaccharide dissolved in D_2_O (Gtf1 and LEUM1747) or in 1 M NaOD in D_2_O (Gtf2 and LEUM1752). Glucans of Gtf2 and LEUM1752 were dissolved by sonication at 100 W and 42 kHz for 30 min. Moreover, δ 4.70 and δ 4.75 ppm of D_2_O and NaOD^1^H signals were used as internal references.

### Methylation analysis

Glucans were solubilized in dry dimethyl sulfoxide (DMSO) by sonication (100 W and 50 kHz for 30 min) and vortexing and were permethylated using methyl iodide and NaOH. After hydrolysis with a mixture of HCl-water-acetic acid and microwave heating, the samples were reduced with NaBD_4_ and then acetylated with a mixture of acetic anhydride-pyridine. The partially methylated alditol acetates obtained were analyzed by GC-MS (GCMS-QP2010; Shimadzu Corp.) with a capillary column (0.25 mm id, 0.25 µm df, 30 m length, DB-5 MS; Agilent Technologies Inc., Santa Clara, CA, USA) programmed to 80°C (2 min), 180°C (20°C/min), and 240°C (4°C/min).

### Production and purification of NTM048 EPS

As described in our earlier study,^16^ NTM048 EPS was extracted and purified. Briefly, 500 µl of an overnight bacterial culture of strain NTM048 was used to inoculate 50 ml of EPS production medium containing 15% sucrose, 0.5% bacto-peptone, 0.5% yeast extract, 1.5% K_2_HPO_4_, 0.001% MnCl_2_·H_2_O, 0.001% NaCl, and 0.005% CaCl_2_, and the culture fluid was incubated for 24 h at 30°C. After the microorganisms were removed by centrifugation, the culture supernatant was precipitated by the addition of an equal volume of chilled ethanol, shaken vigorously, and centrifuged at 8,000 × *g* for 10 min. The supernatant was decanted, and this step was repeated twice. The precipitated EPS was then moderately sonicated to dissolve it in distilled water, followed to purification using Sepharose CL6B (Sigma–Aldrich Corp.) and freeze-drying. The absence of proteins in the EPS sample was confirmed by measuring the absorbance at 280 nm.

### Production and purification of fructan synthesized by LvnS

Fructan, a component of NTM048 EPS, was synthesized by recombinant enzyme LvnS expressed in *E. coli* as described in our earlier study.^27^ The reaction was conducted in 50 mM sucrose, 1 mM CaCl_2_, and 100 mM sodium acetate (pH 5.0) using the recombinant LvnS at 28°C for 24 h. The fructan produced was purified using Sepharose CL6B (Sigma–Aldrich Corp.), and freeze-dried. The absence of proteins in the fructan sample was confirmed by measuring the absorbance at 280 nm.

## In vitro *analysis of the IgA-inducing ability on Peyer’s patch cells*

To evaluate the IgA-inducing ability of polysaccharides, murine PP cells were prepared as described in our earlier study^23^ and then they were re-suspended to reach at 2.5 × 10^6^ cells/ml in complete RPMI 1640 medium (RPMI 1640 [Gibco BRL, Grand Island, NY, USA] containing 100 U/ml penicillin, 100 µg/ml streptomycin, 55 µM 2-mercaptoethanol, and 10% fetal bovine serum [FBS; Gibco BRL]). Various polysaccharides (250 µg/ml final concentration) were added to PP cells (1.25 × 10^6^ cells/ml final concentration) and incubated in 96-well T-cell activation plates (Becton Dickinson, Franklin Lakes, NJ, USA) at 37°C in a humidified atmosphere of 5% CO_2_ in air. After 5 days, the IgA levels in the supernatants were measured using a mouse IgA ELISA kit (Bethyl Laboratories Inc., Montgomery, TX, USA). Data are expressed as a fold change for each polysaccharide relative to LPS (5 µg/ml), for which IgA-inducing activity was normalized to a value of 1.

### Gene expression analysis in bone marrow-derived dendritic cells

BMDCs were generated using murine bone marrow cells as described in our earlier study.^26^ BMDCs were incubated in a complete RPMI 1640 medium at 1.0 × 10^6^ cells/well (3 ml) with or without stimulation by polysaccharides (300 µg/well). After 12 h of stimulation, total RNA was isolated from BMDCs using a QuickPrep Total RNA extraction kit (GE Healthcare), and cDNA was synthesized from the total RNA using a SuperScript III Reverse Transcription kit (Invitrogen, Carlsbad, CA, USA). Real-time PCR was performed using StepOne real-time PCR system (Applied Biosystems, Carlsbad, CA, USA) using a Power SYBR Green Master Mix (Life Technologies Japan, Tokyo, Japan) and primers listed in Table S2. The glyceraldehyde 3-phosphate dehydrogenase (GAPDH) gene was used as an internal control.

### Vaccination of mice

Eight-week-old female BALB/cA mice (CLEA Japan Inc., Tokyo, Japan) were intranasally immunized six times, 7 days apart, with 10 µl of a solution containing 100 µg of glucans (Gtf2-S-glucan or NTM048 EPS) and 5 µg of OVA. Seven days after the last immunization, the mice were euthanized using a mixed anesthetic agent. Plasma, BALF, and nasal wash samples were collected to measure the levels of anti-OVA-specific antibodies. Nasal wash fluid and BALF were collected, respectively, using 200 µl and 1.2 ml of saline. Microtiter plates (MaxiSorp; Nunc, Roskilde, Denmark) were coated with OVA (1 mg/ml), and an ELISA assay was performed. A series of 1/2 dilutions of different samples was loaded onto microtiter plates, and horseradish peroxidase-labeled anti-mouse IgG or IgA (Bethyl Laboratories Inc.) was added to each well. After washing, the microtiter plates were incubated for 15 min with tetramethylbenzidine substrate (Promega Corp., Madison, WI, USA). After the reaction was stopped by adding 0.5 M HCl, absorbance was measured at 450 nm. Animals were handled in accordance with the Guidelines for the Proper Conduct of Animal Experiments issued by the Science Council of Japan (2006). The experimental protocol was approved by the Animal Experimentation Ethics Committee of Ishikawa Prefectural University (approval ID: R2-14-20).

### Statistical analysis

Results are expressed as the mean ± SEM. Data were analyzed using one-way analysis of variance (ANOVA) followed by Tukey–Kramer multiple comparison tests.

## Supplementary Material

Supplemental Material

## Data Availability

The nucleotide sequence data reported in this paper have been deposited in the NCBI database (BOPR01000001).

## References

[1] Zannini E, Waters DM, Coffey A, Arendt EK. Production, properties, and industrial food application of lactic acid bacteria-derived exopolysaccharides. Appl Microbiol Biotechnol. 2015;100(3):1121–17. doi:10.1007/s00253-015-7172-2. 26621802

[2] Saadat YR, Khosroushahi AY, Gargari BP. A comprehensive review of anticancer, immunomodulatory and health beneficial effects of the lactic acid bacteria exopolysaccharides. Carbohydr Polym. 2019;217:79–89. doi:10.1016/j.carbpol.2019.04.025. 31079688

[3] Zhou Y, Cui Y, Qu X. Exopolysaccharides of lactic acid bacteria: structure, bioactivity and associations: a review. Carbohydr Polym. 2019;207:317–332. doi:10.1016/j.carbpol.2018.11.093. 30600013

[4] Welman AD, Maddox IS. Exopolysaccharides from lactic acid bacteria: perspectives and challenges. Trends Biotechnol. 2003;21(6):269–274. doi:10.1016/S0167-7799(03)00107-0. 12788547

[5] Wang K, Li W, Rui X, Chen X, Jiang M, Dong M. Structural characterization and bioactivity of released exopolysaccharides from *Lactobacillus plantarum* 70810. Int J Biol Macromol. 2014;67:71–78. doi:10.1016/j.ijbiomac.2014.02.056. 24631548

[6] Wang J, Zhao X, Yang Y, Zhao A, Yang Z. Characterization and bioactivities of an exopolysaccharide produced by *Lactobacillus plantarum* YW32. Int J Biol Macromol. 2015;74:119–126. doi:10.1016/j.ijbiomac.2014.12.006. 25532782

[7] Li W, Tang W, Ji J, Xia X, Rui X, Chen X, Jiang M, Zhou J, Dong M. Characterization of a novel polysaccharide with anti-colon cancer activity from *Lactobacillus helveticus* MB2-1. Carbohydr Res. 2015;411:6–14. doi:10.1016/j.carres.2014.12.014. 25942063

[8] Du R, Qiao X, Zhao F, Song Q, Zhou Q, Wang Y, Pan L, Han Y, Zhou Z. Purification, characterization and antioxidant activity of dextran produced by *Leuconostoc pseudomesenteroides* from homemade wine. Carbohydr Polym. 2018;198:529–536. doi:10.1016/j.carbpol.2018.06.116. 30093031

[9] Polak-Berecka M, Wasko A, Szwajgier D, Choma A. Bifidogenic and antioxidant activity of exopolysaccharides produced by *Lactobacillus rhamnosus* E/N cultivated on different carbon sources. Pol J Microbiol. 2013;62(2):81–189. doi:10.33073/pjm-2013-023. 24053021

[10] Adesulu-Dahunsi AT, Sanni AI, Jeyaram K. Production, characterization and In vitro antioxidant activities of exopolysaccharide from *Weissella cibaria* GA44. LWT. 2018;87:432–442. doi:10.1016/j.lwt.2017.09.013.

[11] Bhat B, Bajaj BK. Hypocholesterolemic and bioactive potential of exopolysaccharide from a probiotic *Enterococcus faecium* K1 isolated from kalarei. Bioresour Technol. 2018;254:264–267. doi:10.1016/j.biortech.2018.01.078. 29413932

[12] Nakajima H, Suzuki Y, Hirota T. Cholesterol lowering activity of ropy fermented milk. J Food Sci. 1992;57(6):1327–1329. doi:10.1111/j.1365-2621.1992.tb06848.x.

[13] Korcz E, Kerényi Z, Varga L. Dietary fibers, prebiotics, and exopolysaccharides produced by lactic acid bacteria: potential health benefits with special regard to cholesterol-lowering effects. Food Funct. 2018;9(6):3057–3068. doi:10.1039/C8FO00118A. 29790546

[14] Dinić M, Pecikoza U, Djokić J, Stepanović-Petrović R, Milenković M, Stevanović M, Filipović N, Begović J, Golić N, Lukić J. Exopolysaccharide produced by probiotic strain *Lactobacillus paraplantarum* BGCG11 reduces inflammatory hyperalgesia in rats. Front Pharmacol. 2018;9:1. doi:10.3389/fphar.2018.00001. 29387012 PMC5776101

[15] Matsuzaki C, Hayakawa A, Matsumoto K, Katoh T, Yamamoto K, Hisa K. Exopolysaccharides produced by *Leuconostoc mesenteroides* strain NTM048 as an immunostimulant to enhance the mucosal barrier and influence the systemic immune response. J Agric Food Chem. 2015;63(31):7009–7015. doi:10.1021/acs.jafc.5b01960. 26207929

[16] Matsuzaki C, Takagaki C, Tomabechi Y, Forsberg LS, Heiss C, Azadi P, Matsumoto K, Katoh T, Hosomi K, Kunisawa J, et al. Structural characterization of the immunostimulatory exopolysaccharide produced by *Leuconostoc mesenteroides* strain NTM048. Carbohydr Res. 2017;448:95–102. doi:10.1016/j.carres.2017.06.004. 28633071

[17] Ren W, Xia Y, Wang G, Zhang H, Zhu S, Ai L. Bioactive exopolysaccharides from a *S. thermophilus* strain: screening, purification and characterization. Int J Biol Macromol. 2016;86:402–407. doi:10.1016/j.ijbiomac.2016.01.085. 26820354

[18] Ferreira SS, Passos CP, Madureira P, Vilanova M, Coimbra MA. Structure–function relationships of immunostimulatory polysaccharides: a review. Carbohydr Polym. 2015;132:378–396. doi:10.1016/j.carbpol.2015.05.079. 26256362

[19] Lombard V, Golaconda Ramulu H, Drula E, Coutinho PM, Henrissat B. The carbohydrate-active enzymes database (CAZy) in 2013. Nucleic Acids Res. 2014;42(D1):D490–D495. doi:10.1093/nar/gkt1178. 24270786 PMC3965031

[20] Korakli M, Vogel RF. Structure/function relationship of homopolysaccharide producing glycansucrases and therapeutic potential of their synthesised glycans. Appl Microbiol Biotechnol. 2006;71(6):790–803. doi:10.1007/s00253-006-0469-4. 16724190

[21] Ryan PM, Ross RP, Fitzgerald GF, Caplice NM, Stanton C. Sugar-coated: exopolysaccharide producing lactic acid bacteria for food and human health applications. Food Funct. 2015;6(3):679–693. doi:10.1039/C4FO00529E. 25580594

[22] Torino MI, Font De Valdez G, Mozzi F. Biopolymers from lactic acid bacteria. Novel applications in foods and beverages. Front Microbiol. 2015;6:834. doi:10.3389/fmicb.2015.00834. 26441845 PMC4566036

[23] Matsuzaki C, Kamishima K, Matsumoto K, Koga H, Katayama T, Yamamoto K, Hisa K. Immunomodulating activity of exopolysaccharide‐producing *Leuconostoc mesenteroides* strain NTM 048 from green peas. J Appl Microbiol. 2014;116(4):980–989. doi:10.1111/jam.12411. 24314091

[24] Fagarasan S, Honjo T. Intestinal IgA synthesis: regulation of front-line body defences. Nat Rev Immunol. 2003;3(1):63–72. doi:10.1038/nri982. 12511876

[25] Tezuka H, Ohteki T. Regulation of IgA production by intestinal dendritic cells and related cells. Front Immunol. 2019;10:1891. doi:10.3389/fimmu.2019.01891. 31456802 PMC6700333

[26] Matsuzaki C, Takagaki C, Higashimura Y, Nakashima Y, Hosomi K, Kunisawa J, Yamamoto K, Hisa K. Immunostimulatory effect on dendritic cells of the adjuvant-active exopolysaccharide from *Leuconostoc mesenteroides* strain NTM048. Biosci Biotechnol Biochem. 2018;82(9):1647–1651. doi:10.1080/09168451.2018.1482195. 29863431

[27] Ishida R, Sakaguchi K, Matsuzaki C, Katoh T, Ishida N, Yamamoto K, Hisa K. Levansucrase from *Leuconostoc mesenteroides* NTM048 produces a levan exopolysaccharide with immunomodulating activity. Biotechnol Lett. 2016;38(4):681–687. doi:10.1007/s10529-015-2024-9. 26960415

[28] van Hijum SA, Kralj S, Ozimek LK, Dijkhuizen L, van Geel-Schutten IG. Structure-function relationships of glucansucrase and fructansucrase enzymes from lactic acid bacteria. Microbiol Mol Biol Rev. 2006;70(1):157–176. doi:10.1128/MMBR.70.1.157-176.2006. 16524921 PMC1393251

[29] Vujičić-Žagar A, Pijning T, Kralj S, López CA, Eeuwema W, Dijkhuizen L, Dijkstra BW. Crystal structure of a 117 kDa glucansucrase fragment provides insight into evolution and product specificity of GH70 enzymes. Proc Natl Acad Sci USA. 2010;107(50):21406–21411. doi:10.1073/pnas.1007531107. 21118988 PMC3003066

[30] Li X, Wang X, Meng X, Dijkhuizen L, Liu W. Structures, physico-chemical properties, production and (potential) applications of sucrose-derived α-D-glucans synthesized by glucansucrases. Carbohydr Polym. 2020;249:116818. doi:10.1016/j.carbpol.2020.116818. 32933666

[31] Matsuzaki C, Matsumoto K, Katoh T, Yamamoto K, Hisa K. Comparison of activity to stimulate mucosal IgA production between *Leuconostoc mesenteroides* strain NTM048 and type strain JCM6124 in mice. Biosci Microbiota Food Health. 2016;35(1):51–55. doi:10.12938/bmfh.2015-020. 26858930 PMC4735033

[32] Monchois V, Remaud-Simeon M, Russell RRB, Monsan P, Willemot RM. Characterization of *Leuconostoc mesenteroides* NRRL B-512F dextransucrase (DSRS) and identification of amino-acid residues playing a key role in enzyme activity. Appl Microbiol Biotechnol. 1997;48(4):465–472. doi:10.1007/s002530051081. 9390454

[33] Funane K, Ishii T, Matsushita M, Hori K, Mizuno K, Takahara H, Kitamura Y, Kobayashi M. Water-soluble and water-insoluble glucans produced by *Escherichia coli* recombinant dextransucrases from *Leuconostoc mesenteroides* NRRL B-512F. Carbohydr Res. 2001;334(1):19–25. doi:10.1016/S0008-6215(01)00163-X. 11470247

[34] Claverie M, Cioci G, Vuillemin M, Monties N, Roblin P, Lippens G, Remaud-Simeon M, Moulis C. Investigations on the determinants responsible for low molar mass dextran formation by DSR-M dextransucrase. ACS Catal. 2017;7(10):7106–7119. doi:10.1021/acscatal.7b02182.

[35] Kang HK, Kim YM, Kim DM. Functional, genetic, and bioinformatic characterization of dextransucrase (DSRBCB4) gene in *Leuconostoc mesenteroides* B-1299CB4. J Microbiol Biotechnol. 2008;18:1050–1058. 18600046

[36] Wangpaiboon K, Waiyaseesang N, Panpetch P, Charoenwongpaiboon T, Nepogodiev SA, Ekgasit S, Field RA, Pichayangkura R. Characterisation of insoluble α-1, 3-/α-1, 6 mixed linkage glucan produced in addition to soluble α-1, 6-linked dextran by glucansucrase (DEX-N) from *Leuconostoc citreum* ABK-1. Int J Biol Macromol. 2020;152:473–482. doi:10.1016/j.ijbiomac.2020.02.247. 32097735

[37] Kim D, Robyt JF. Production and selection of mutants of *Leuconostoc mesenteroides* constitutive for glucansucrases. Enzyme Microb Technol. 1994;16(8):659–664. doi:10.1016/0141-0229(94)90086-8. 7519863

[38] Zahnley JC, Smith MR. Insoluble Glucan Formation by *Leuconostoc mesenteroides* B-1355. Appl Environ Microbiol. 1995;61(3):1120–1123. doi:10.1128/aem.61.3.1120-1123.1995. 16534961 PMC1388393

[39] Jeanes A, Haynes WC, Wilham CA, Rankin JC, Melvin EH, Austin MJ, Cluskey JE, Fisher BE, Tsuchiya HM, Rist CE. Characterization and classification of dextrans from ninety-six strains of bacteria. J Am Chem Soc. 1954;76(20):5041–5052. doi:10.1021/ja01649a011.

[40] Funane K, Mizuno K, Takahara H, Kobayashi M. Gene encoding a dextransucrase-like protein in *Leuconostoc mesenteroides* NRRL B-512F. Biosci Biotechnol Biochem. 2000;64(1):29–38. doi:10.1271/bbb.64.29. 10705445

[41] Ruas-Madiedo P. Biosynthesis and bioactivity of exopolysaccharides produced by probiotic bacteria. In: Moreno FJ, Sanz ML, editors. Food Oligosaccharides. Chichester (UK): John Wiley & Sons; 2014. p. 118–133.

[42] Kitazawa H, Harata T, Uemura J, Saito T, Kaneko T, Itoh T. Phosphate group requirement for mitogenic activation of lymphocytes by an extracellular phosphopolysaccharide from *Lactobacillus delbrueckii* ssp. *bulgaricus*. Int J Food Microbiol. 1998;40(3):169–175. doi:10.1016/S0168-1605(98)00030-0. 9620124

[43] Sato T, Nishimura-Uemura J, Shimosato T, Kawai Y, Kitazawa H, Saito T. Dextran from *Leuconostoc mesenteroides* augments immunostimulatory effects by the introduction of phosphate groups. J Food Prot. 2004;67(8):1719–1724. doi:10.4315/0362-028X-67.8.1719. 15330539

[44] Zeidan AA, Poulsen VK, Janzen T, Buldo P, Derkx PM, Øregaard G, Neves AR. Polysaccharide production by lactic acid bacteria: from genes to industrial applications. FEMS Microbiol Rev. 2017;41(Supp_1):S168–S200. doi:10.1093/femsre/fux017. 28830087

[45] Hidalgo-Cantabrana C, López P, Gueimonde M, Clara G, Suárez A, Margolles A, Ruas-Madiedo P. Immune modulation capability of exopolysaccharides synthesised by lactic acid bacteria and bifidobacteria. Probiotics Antimicrob Proteins. 2012;4(4):227–237. doi:10.1007/s12602-012-9110-2. 26782182

[46] Bleau C1, Monges A, Rashidan K, Jp L, Lacroix M, Van Calsteren MR, Millette M, Savard R, Lamontagne L. Intermediate chains of exopolysaccharides from *Lactobacillus rhamnosus* RW‐9595M increase IL‐10 production by macrophages. J Appl Microbiol. 2010;108(2):666–675. doi:10.1111/j.1365-2672.2009.04450.x. 19702865

[47] Lopez P, Monteserin DC, Gueimonde M, Clara G, Margolles A, Suarez A, Ruas-Madiedo P. Exopolysaccharide-producing *Bifidobacterium* strains elicit different in vitro responses upon interaction with human cells. Food Res Int. 2012;46(1):99–107. doi:10.1016/j.foodres.2011.11.020.

[48] Aderem A, Underhill DM. Mechanisms of phagocytosis in macrophages. Annu Rev Immunol. 1999;17(1):593–623. doi:10.1146/annurev.immunol.17.1.593. 10358769

[49] Yameen B, Choi WI, Vilos C, Swami A, Shi J, Farokhzad OC. Insight into nanoparticle cellular uptake and intracellular targeting. J Control Release. 2014;190:485–499. doi:10.1016/j.jconrel.2014.06.038. 24984011 PMC4153400

[50] Fujioka-Hirai Y, Akagawa Y, Minagi S, Tsuru H, Miyake Y, Suginaka H. Adherence of *Streptococcus mutans* to implant materials. J Biomed Mater Res. 1987;21(7):913–920. doi:10.1002/jbm.820210707. 3038919

[51] Kang X, Kirui A, Muszyński A, Widanage MCD, Chen A, Azadi P, Wang P, Mentink-Vigier F, Wang T. Molecular architecture of fungal cell walls revealed by solid-state NMR. Nat Commun. 2018;9(1):1–12. doi:10.1038/s41467-018-05199-0. 30013106 PMC6048167

[52] Hatayama T, Segawa R, Mizuno N, Eguchi S, Akamatsu H, Fukuda M, Nakata F, Leonard WJ, Hiratsuka M, Hirasawa N. All-trans retinoic acid enhances antibody production by inducing the expression of thymic stromal lymphopoietin protein. J Immunol. 2018;200(8):2670–2676. doi:10.4049/jimmunol.1701276. 29500243

[53] Thompson JD, Gibson TJ, Higgins DG. Multiple sequence alignment using ClustalW and ClustalX. Curr Protoc Bioinformatics. 2003;1:2.3.1–2.3.22. 10.1002/0471250953.bi0203s00 18792934

[54] Kumar S, Stecher G, Li M, Knyaz C, Tamura K. MEGA X: molecular Evolutionary Genetics Analysis across computing platforms. Mol Biol Evol. 2018;35:1547–1549. doi:10.1093/molbev/msy096. 29722887 PMC5967553

[55] Saitou N, Nei M. The neighbor-joining method: a new method for reconstructing phylogenetic trees. Mol Biol Evol. 1987;4:406–425. doi:10.1093/oxfordjournals.molbev.a040454. 3447015

[56] Felsenstein J. Confidence limits on phylogenies: an approach using the bootstrap. Evol. 1985;39:783–791. doi:10.1111/j.1558-5646.1985.tb00420.x. 28561359

[57] Sumner JB, Howell SF. A method for determination of saccharase activity. J Biol Chem. 1935;108(1):51–54. doi:10.1016/S0021-9258(18)75307-6.

