# Supplementary material for: Enzymatically synthesized exopolysaccharide of a probiotic strain Leuconostoc mesenteroides NTM048 shows adjuvant activity to promote IgA antibody responses
Source: Gut Microbes. 2021 Jul 21;13(1):1949097. doi: 10.1080/19490976.2021.1949097 (PMC8550178; doi:10.1080/19490976.2021.1949097)
Supplement: Supplemental Material [file KGMI_A_1949097_SM5910.zip › supplementary/Supporting information final.docx]

**Supporting information**

Table S1. Oligonucleotides used for cloning.

|  | Primer name | Sequence |
| --- | --- | --- |
| *gtf1* cloning primers | 23b_IF_gtf1-F | 5’-AAGGAGATATACATATGacaccaatgttttg-3’ |
|  | 23b_IF_gtf1-R | 5’-TGCTCGAGTGCGGCCGCtgctgacacagcatt-3’ |
|  |  |  |
| *gtf2* cloning primers | 23b_IF_gtf2-F | 5’-AAGGAGATATACATATGgatgtttcacaaaac-3’ |
|  | 23b_IF_gtf2-R | 5’-TGCTCGAGTGCGGCCGCgcttttaatcagctc-3’ |
|  |  |  |
| *LEUM1742* cloning primers | 23b_IF_LEUM1747-F | 5’-AAGGAGATATACATATGacaccaagtgttttg-3’ |
|  | 23b_IF_LEUM1747-R | 5’-TGCTCGAGTGCGGCCGCtgctgacacagcatt-3’ |
|  |  |  |
| *LEUM1752* cloning primers | 23b_IF_LEUM1752-F | 5’-AAGGAGATATACATATGgatgtttcacaaaac-3’ |
|  | 23b_IF_LEUM1752-R | 5’-TGCTCGAGTGCGGCCGCgcttttaatcatctc-3’ |

Restriction enzyme recognition sites are underlined.

Table S2. Oligonucleotides used for gene expression analysis.

| Gene Name | Primer orientation | Sequence |
| --- | --- | --- |
| GAPDH (*Gapdh*) | Forward | 5'-CTACACTGAGGACCAGGTTGTCT-3' |
|  | Reverse | 5'-ATTGTCATACCAGGAAATGAGCTT-3' |
|  |  |  |
| IL-6 (*Il6*) | Forward | 5'-AATAGTCCTTCCTACCCCAATTTC-3' |
|  | Reverse | 5'-ATTTCAAGATGAATTGGATGGTCT-3' |
|  |  |  |
| RALDH2 (*Aldh1a2*) | Forward | 5'-GACTTGTAGCAGCTGTCTTCACT-3' |
|  | Reverse | 5'-TCACCCATTTCTCTCCCATTTCC-3' |


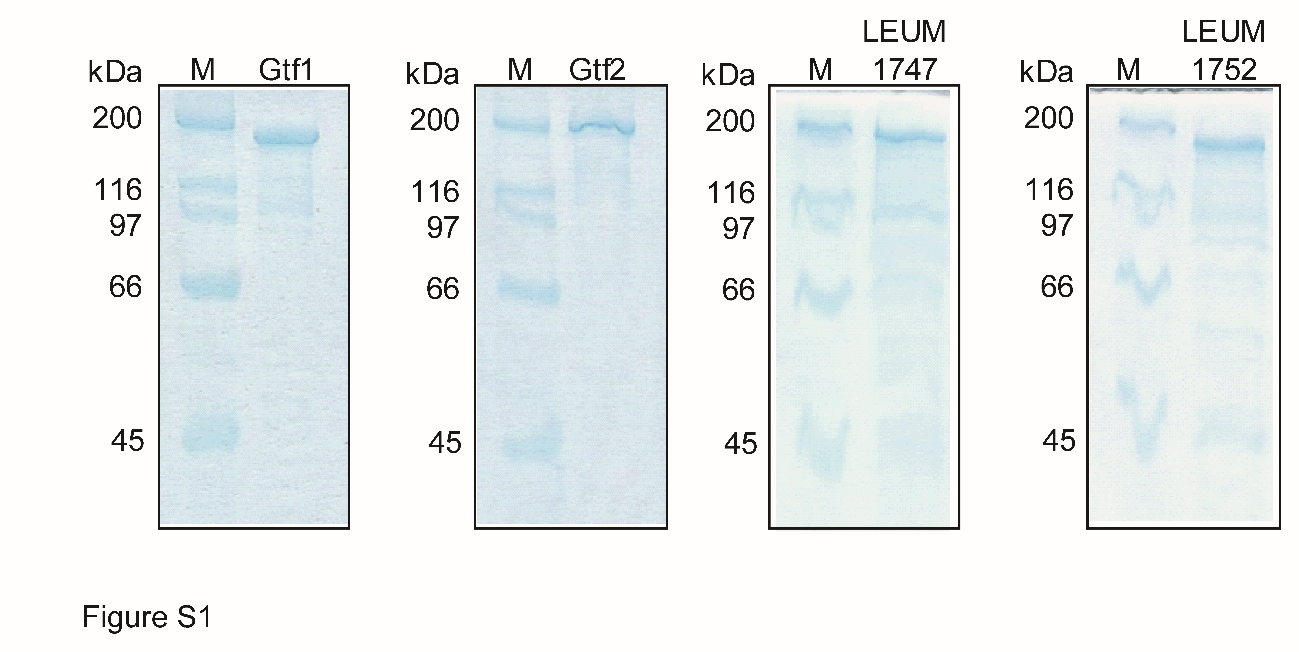


Figure S1. SDS-PAGE analysis of the purified glucosyltransferases: Gtf1and Gtf2 from strain NTM048 and LEUM1747 and LEUM1752 from strain JCM6142. Proteins were expressed in *Escherichia coli* BL21 (DE3) cells and purified by His-tag affinity purification and anion exchange chromatography. M: protein molecular weight marker.
